# Study on Milling Force and Surface Quality during Slot Milling of Plain-Woven CFRP with PCD Tools

**DOI:** 10.3390/ma15113862

**Published:** 2022-05-28

**Authors:** Ziyang Xu, Yongguo Wang

**Affiliations:** School of Mechatronics Engineering and Automation, Shanghai University, Shanghai 200444, China; ziyangxu@shu.edu.cn

**Keywords:** woven CFRP, equivalent cutting area, cutting force, surface roughness, delamination

## Abstract

Carbon-fiber-reinforced polymers (CFRPs) have been widely used in many industrial fields, such as automobile, aerospace and so on, because of their excellent mechanical properties. However, due to their anisotropy and inhomogeneity, machining CFRPs is a great challenge. In this paper, the slot milling of a plain-woven CFRP with PCD tools is carried out, and the effects of cutting parameters and tool rake angle on cutting force and surface roughness are studied. The results show that the 4° rake angle PCD tool has smaller cutting force than the 0° rake angle PCD tool, but the effect of rake angle on surface roughness is not significant. The concept of equivalent cutting area is introduced to study the variation law of cutting force and surface roughness. It is found that the cutting force and surface roughness increase with the increase in equivalent cutting area, and decrease with the decrease in equivalent cutting area. The removal mechanism of surface materials under different equivalent cutting areas is different, which leads to the difference in surface roughness. Finally, the causes of delamination on the top layer after milling are explained.

## 1. Introduction

Carbon-fiber-reinforced polymers (CFRPs) are widely used in the aviation, aerospace, automotive and defense industries due to their high specific strength, high specific stiffness, good fatigue resistance and excellent fracture toughness [1,2,3,4]. In order to avoid rough machining operations, CFRP components are generally produced by a near-net-shape method [5], but production processes such as milling or drilling must be performed to remove excess material in order to make the parts meet the dimensional tolerance and quality requirements [6].

However, due to the different mechanical properties between the phases of CFRPs, the material has the characteristics of inhomogeneity and anisotropy [7]. Different from the plastic deformation when processing metal alloys, CFRPs almost only exhibit brittle fracture during processing, so various defects, such as fiber pull-out, fiber fracture and delamination, appear easily during processing [8,9]. Therefore, processing high-quality CFRP parts is a huge challenge in the industry.

Cutting force can usually be used to evaluate the cutting state. Excessive milling force will aggravate the vibration between the tool and the workpiece, resulting in defects on the surface of the workpiece and quality problems. Therefore, the milling force needs to be strictly controlled during the machining process. A lot of literature has studied the cutting force in milling unidirectional CFRPs. Lifeng Zhang et al. [10] studied the milling force at four typical fiber-orientation angles, and found that the cutting force was the largest at 90°, followed by the angle of 135°, and the smallest cutting force appeared at the orientation angle of 0°. Jia Zhenyuan [11] also found that the cutting force increases slowly in the range of 0–45°, and the increase rate is faster in the range of 45–135°. V Madhavan [12] conducted vertical cutting experiments on the outer diameter of unidirectional CFRP-laminate discs to further study the changes in cutting force data when continuously cutting CFRPs at a 0–90° fiber orientation angle (FOA). The results show that, for large feeds, the cutting force increases with increasing FOA until FOA = 90°, and for low feeds, the maximum cutting force appears at FOA = 65°, and then gradually decreases.

Some scholars focus on studying the surface quality of processed CFRPs. Wang C. [13] explained that the formation of cavities on the surface of unidirectional CFRPs is mainly due to the occurrence and expansion of fiber and matrix debonding, followed by fiber fracture caused by bending and shearing. Researchers have also discussed the influence of cutting parameters on the quality of machined surfaces. El-Hofy et al. [14] found that low cutting speed and high feed rate are the best processing conditions for CFRP grooving and milling. Çolak and Sunar [15] concluded, on the contrary, that an increase in cutting speed and a decrease in feed speed would lead to better surface quality. Although Nguyen-Dinh et al. [16] proposed new surface quality criteria, namely crater volume (Cv) and depth of damage (D), to describe the machining damage, more scholars [17,18,19] still prefer to use Ra to describe the machined-surface quality.

Muhamad KNK [20] has found that the abrasiveness of carbon fibers will lead to excessive wear of the tool when machining CFRP materials with cemented carbide tools. However, Nguyen D N [21] found that when the machining distance of CFRPs with a PCD tool reached 1.68 m, the maximum tool wear was only 11 μm. In addition, more literature [22,23,24] shows that machining CFRPs with a PCD tool not only results in low tool wear, but also is more conducive to obtaining better surface quality.

The research on plain-woven CFRPs is more focused on drilling [25,26,27], and only a small amount of literature studies the milling of woven CFRPs. Hintze et al. [28] studied the effect of the position of trimming edge on the dispersion of woven CFRPs. They found that the thickness of the resin layer on the top will change with the fluctuation of the fiber, which will lead to two quality defects: fiber protrusion and surface damage. The research also shows that the influence of tool geometry on workpiece delamination is not significant. Li Maojun [29] compared the cutting force and surface quality when milling multidirectional CFRPs and woven CFRPs. It was found that the cutting force in milling woven CFRPs is greater than that in multidirectional CFRPs, but the surface roughness is smaller.

In this study, a plain-woven CFRP was used for milling research. PCD tools with rake angles 0°and 4° were used to slot the milling of the woven CFRP. The cutting forces in the milling process were collected, and the surface roughness was measured after machining. The variation law of cutting force and surface roughness under different equivalent chip areas was studied. In addition, the machined surface of a PCD tool with 0° rake angle was observed using an electron scanning microscope, and the material removal mechanism under different equivalent cutting areas was revealed.

## 2. Preparation Works

### 2.1. Workpiece Materials and Cutting Tools

**Figure 1:**
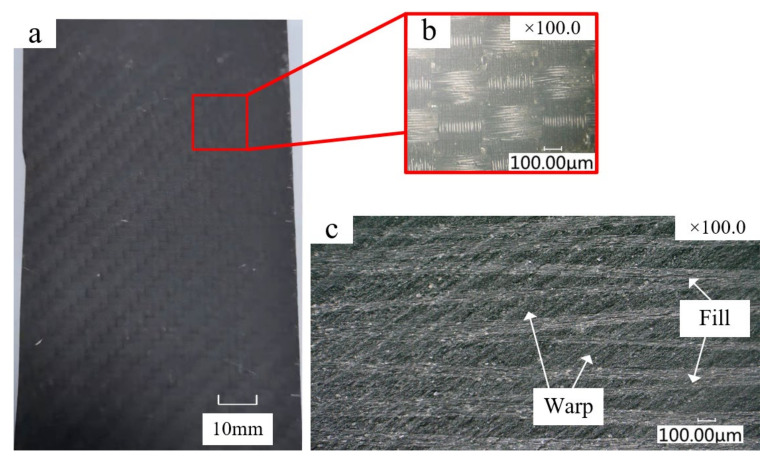
Structure of the woven CFRP. (**a**) CFRP material used in the experiment; (**b**) woven structure of the carbon fiber; (**c**) cutaway view of the plain-woven CFRP.

The CFRP laminate was compacted with a vacuum pump and then cured in an autoclave at 180 °C for 120 min. Figure 1 shows the plain-woven CFRP laminate used in the experiment. The fibers are interlaced at 0°/90°. The fiber parallel to the milling feed direction is called the warp fiber, and the vertically oriented fiber is called the weft fiber. The fiber volume content is 60%, and the average diameter of the carbon fibers is 7–8 μm. The average thickness per layer is about 0.2 mm, there are 50 layers in total, and the total thickness is 10 mm. In addition, in order to facilitate clamping and machining, the laminate was divided into 125 × 75 × 10 mm for the slot-milling experiment. Table 1 shows the mechanical properties of the CFRP.

**Figure 2:**
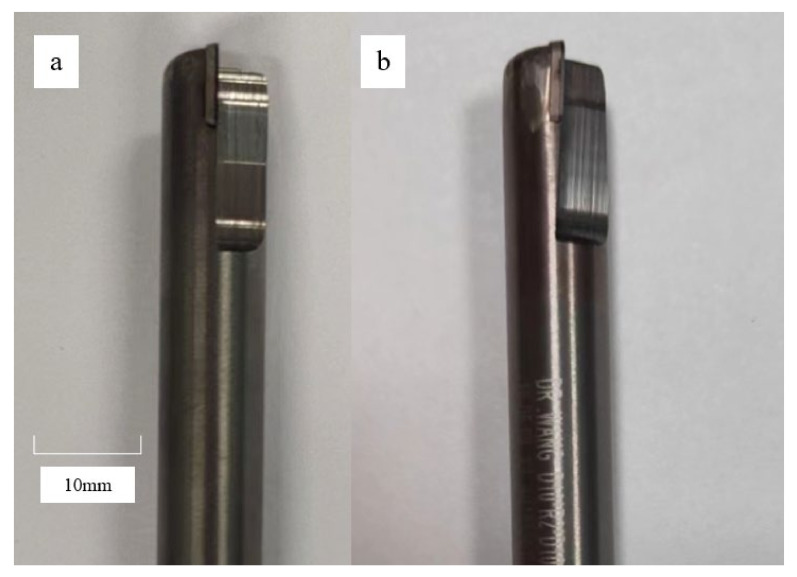
Cutting tools. (**a**) PCD tool with 0° rake angle; (**b**) PCD tool with 4° rake angle.

The PCD tools used in the experiment were provided by Guohong tool system (Wuxi) Co., Ltd., Wuxi, China. Figure 2 shows the two PCD tools used in the experiment, with an average grain size of 10 μm, and Table 2 shows the specific parameters of the tools. 

### 2.2. Equivalent Cutting Area

**Figure 3:**
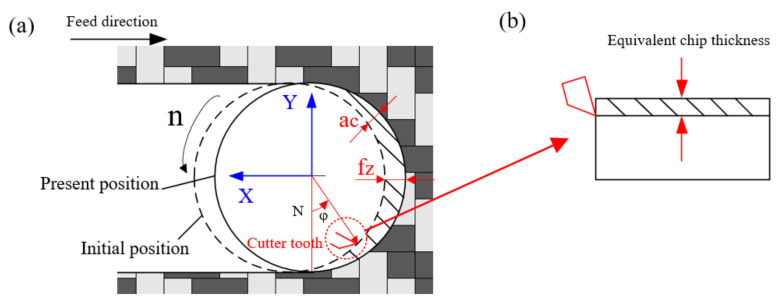
Schematic diagram of equivalent chip. (**a**) Instantaneous milling process; (**b**) Schematic diagram of equivalent chip thickness.

Figure 3a shows the chip schematic diagram during tool milling. The tool feed direction is defined by the *X*-axis, and the direction perpendicular to the feed is defined by the *Y*-axis. $f_{z}$ represents the feed per tooth, $a_{c}$ represents the thickness of the instantaneous uncut chip. The cutter rotation angle φ is measured counterclockwise from the vertical direction. Obviously, the value of $a_{c}$ changes with the rotation angle of the tool [30]. The instantaneous uncut chip thickness can be described as follows:

(1) $$a_{c}=f_{z}\sin\varphi$$

Since the linear velocity of the tool is much greater than its feed rate in the actual milling of the CFRP, the chip-thickness changes are very limited [31]. Therefore, the instantaneous chip can be converted into an equivalent chip with uniform thickness, as shown in Figure 3b. The calculation formula is as follows:

(2) $$a_{eq}=a_{e}\;\frac{V_{f}}{V_{c}}$$

where $a_{e}$ is the radial depth of cut, and in this paper, $a_{e}$ represents the tool diameter, so $a_{e}$ = 10 mm, $V_{f}$ means the feed rate (m/min), $V_{c}$ = π d n is the cutting speed (m/min), d represents the diameter of the milling tool, n is the spindle speed (rpm).

Through the above equivalent method, we can regard the milling process as orthogonal plane cutting. So, the instantaneous cutting area can be written as [32]:

(3) $$\bar{A}=a_{p}\;a_{eq}=a_{p}\;a_{e}\;\frac{V_{f}}{V_{c}}$$

where $\bar{A}$ is the equivalent cutting area, $a_{p}$ means the axial depth of the cut.

In this paper, the three cutting parameters are combined through Equation (3) to study the change in equivalent cutting area, milling force and surface roughness. This is different from the influence of a single variable on milling force and surface quality studied by other scholars.

The cutting experiment contains three variables: spindle speed, axial depth of cut and feed rate, and each variable contain three levels, so it is not appropriate to use the full factor experiment. Therefore, Taguchi orthogonal experimental design is commonly used to solve such situations. The experimental parameters used in this paper and the corresponding equivalent cutting area are shown in Table 3 below. The selection of experimental parameters depends on the actual production experience and processing efficiency.

### 2.3. Experimental Setup and Measuring System

**Figure 4:**
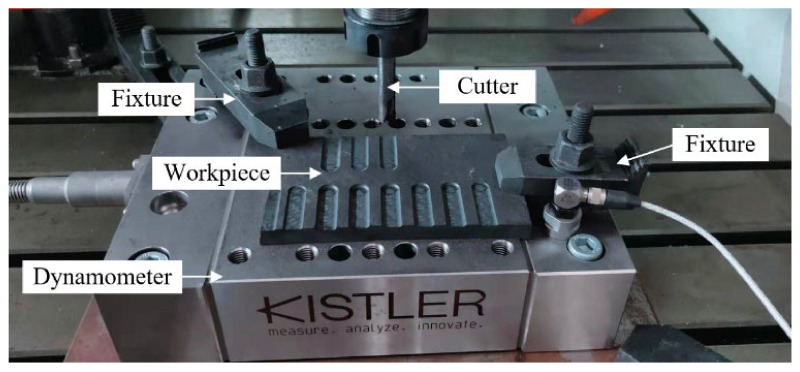
Experimental setup.

The milling tests were carried out on a Carver S600A RT 3-axis CNC vertical machining center with a maximum spindle speed of 20,000 rpm. The cutting forces were collected with a Kistler-9255C dynamometer, and the charge signals obtained by the dynamometer were amplified through Kistler-5167A. Finally, the computer signal acquisition system was used to collect the cutting forces. The experimental setup is shown in Figure 4.

**Figure 5:**
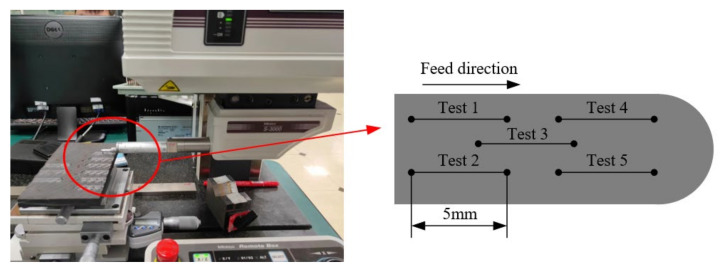
The surface roughness measurement system and measurement method.

The surface roughness of machined surface was measured by using a Mitutoyo S-3000 portable profilometer. The average of five measurements on each surface was taken, in order to eliminate errors to the greatest extent. Figure 5 shows the surface roughness measurement system and measurement method [33]. A Zeiss Evo 18 scanning electron microscope was used to study the microscopic surface morphology after milling and before the SEM. The laminates were cut into small cuboids and cleared by an ultrasonic cleaning device.

In the experiment, each cutter milled a groove on the surface of the composite material with nine different processing parameters, and each groove was 15 mm long. The total distance of the milling process of each tool was 9 × 15 = 135 mm.

## 3. Results and Discussion

### 3.1. Effect of Equivalent Cutting Area on Milling Force

The cutting force signal collected in the milling process is processed by a low-pass filter, and the average value of the stable cutting period is selected as the milling force for correlation analysis. Because the helix angle is zero, the axial cutting force is far less than the cutting force in the X and Y directions, so the calculation of $F_{z}$ is ignored in this paper. Based on the milling force measured in the X and Y directions, the cutting force is calculated by the following equation:

(4) $$F=\sqrt{F_{x}^{2}+F_{y}^{2}}$$

**Figure 6:**
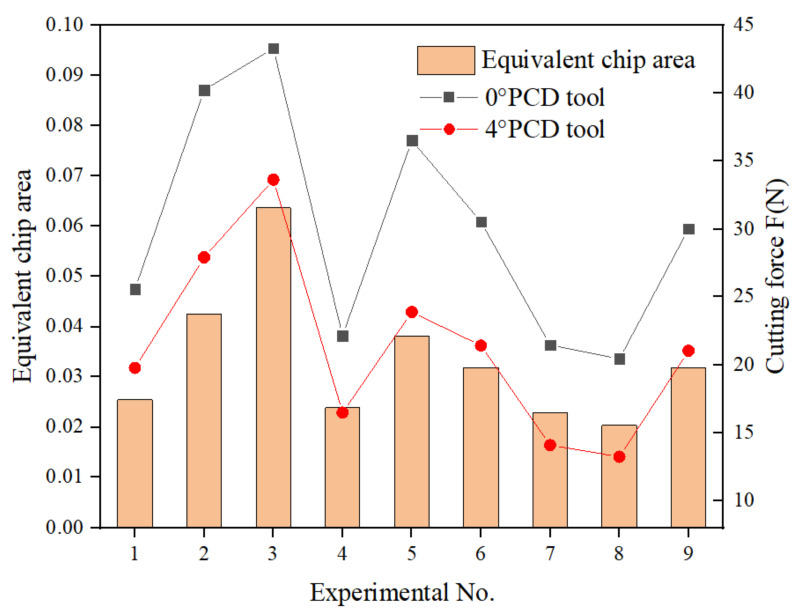
Variation of cutting force with equivalent cutting area.

Figure 6 shows the variation law of the cutting force of the two tools with the equivalent chip area. First of all, it can be seen that the cutting force of the 4° rake PCD tool is less than that of the 0° rake tool. This is because the tool rake angle increases, which not only makes the cutting edge sharper, but also is more conducive to the outflow of chips along the rake face. It can also be seen that the cutting force of both tools is directly proportional to the equivalent chip area, that is, when the equivalent cutting area increases, the cutting force increases, and when the equivalent cutting area decreases, the cutting force will also decrease. When the spindle speed is 6000 r/min, the feed rate is 0.8 m/min and the cutting depth is 1.2 mm, the equivalent chip area is the largest, and the maximum cutting force is observed, which is 43.32 N and 33.63 N, respectively. Under the eighth set of parameters, the equivalent chip area is the smallest, and the minimum cutting force is obtained, which is 20.44 N and 13.22 N, respectively. On the one hand, the reduction in cutting force is due to the reduction in equivalent cutting area. On the other hand, the minimum equivalent cutting area corresponds to the maximum spindle speed. At this time, the temperature will increase with the increase of the speed. When the temperature rises to the glass-transition temperature of the matrix resin, the resin softens and the tool can more easily cut the matrix, so the cutting force is further reduced [34]. It can be seen that, although the equivalent chip area is not the real chip thickness, it can affect the cutting force to a certain extent. Therefore, the cutting force can be reduced by controlling the equivalent chip area.

**Figure 7:**
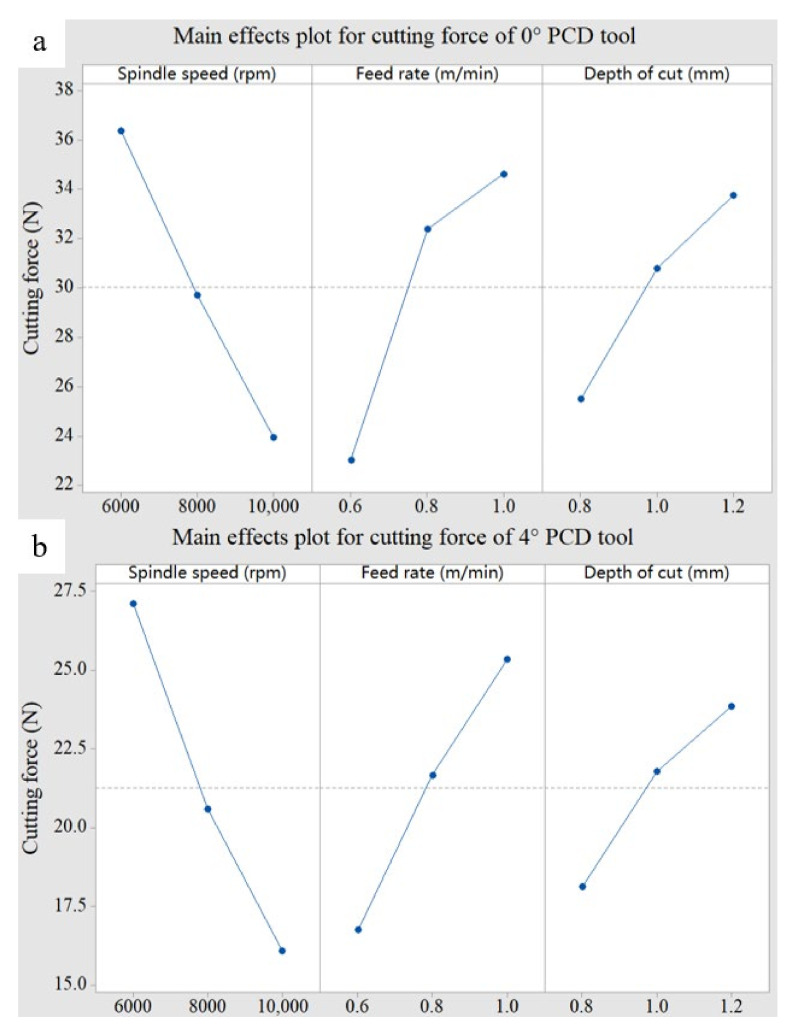
Main effects plot for cutting force. (**a**) 0° PCD tool; (**b**) 4°PCD tool.

The detailed information concerning main effects plots of cutting force related to different cutting parameters is shown in Figure 7. The cutting force decreases with the increase in spindle speed and increases with the increase in feed rate and cutting depth. This can be explained by equivalent cutting area. When the spindle speed increases, the cutting volume per tooth decreases and the equivalent cutting area decreases, so the cutting force will decrease. When the feed rate and cutting depth increase, the equivalent cutting area increases and the cutting force increases. This is consistent with the conclusion of Janardhan P’s [35] research.

The functional relationship between cutting force and equivalent cutting area is established by using a nonlinear fitting method [36], as follows:

(5) $$0{}^{\circ}\;\mathrm{PCD}\;\mathrm{tool}:\;F=388.15\times {\left(a_{p}\;a_{e}\;\frac{V_{f}}{V_{c}}\right)}^{0.75}\;\;\;\;\;\;\;R^{2}=0.926$$

(6) $$4{}^{\circ}\;\mathrm{PCD}\;\mathrm{tool}:\;F=349.94\times {\left(a_{p}\;a_{e}\;\frac{V_{f}}{V_{c}}\right)}^{0.821}\;\;\;\;\;R^{2}=0.931$$

### 3.2. Effect of Equivalent Cutting Area on Surface Roughness

**Figure 8:**
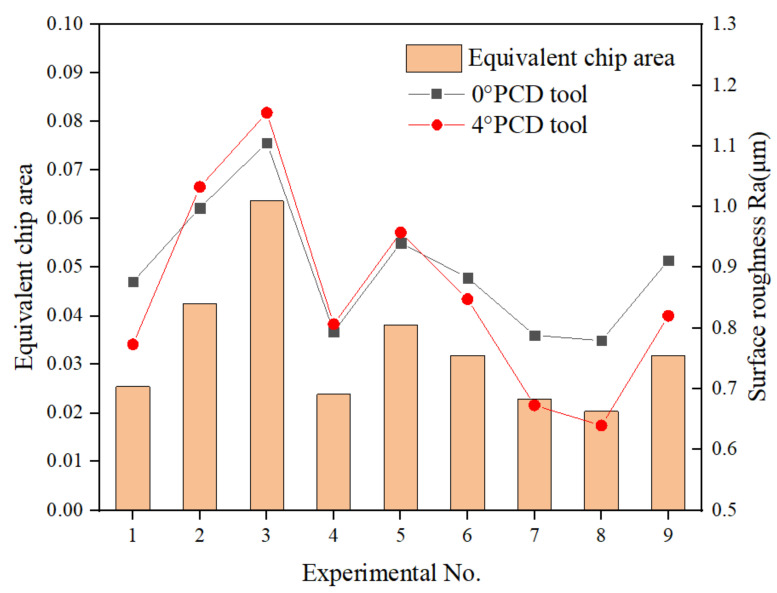
Variation of surface roughness with equivalent cutting area.

**Figure 9:**
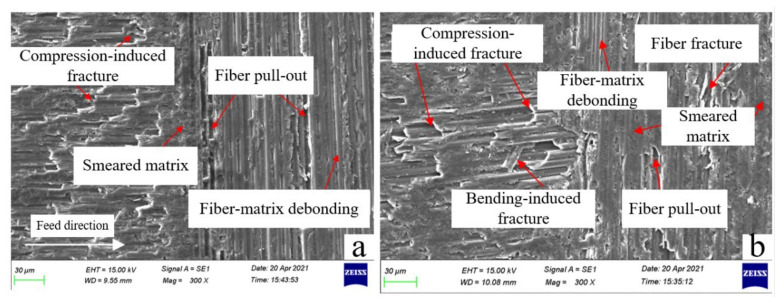
Micrographs of machined surface; (**a**) the maximum equivalent cutting area (n = 6000 r/min, $V_{f}$ = 1 m/min, $a_{p}$ = 1.2 mm); (**b**) the minimum equivalent cutting area (*n* = 10,000 r/min, $V_{f}$ = 0.8 m/min, $a_{p}$ = 0.8 mm).

Figure 8 shows the variation law of surface roughness with equivalent chip area. It can be seen that the roughness of the machined surface does not depend on the tool geometry, but on the cutting parameters. The surface roughnesses of two cutting tools are both positively correlated with the equivalent cutting area [37]. Under the third set of parameters, the equivalent cutting area is the largest, and the surface roughnesses after machining by the two PCD tools reach their maximums at this time, which are 1.1053 μm and 1.1548 μm, respectively. When the equivalent cutting area is the smallest, the surface roughnesses of the two tools also achieve their minimum values, which are 0.7794 μm and 0.6395 μm, respectively. This phenomenon can be explained by different cutting mechanisms under different equivalent cutting areas. Figure 9a,b shows the micromorphology of the 0° PCD tool under the maximum and minimum equivalent chip areas, respectively. It can be seen that when the tool is milling under the maximum equivalent cutting area, the carbon-fiber-removal mode parallel to the feed direction is mainly the fracture caused by compression, while in the direction perpendicular to the tool feed direction, fiber-matrix debonding and fiber pull-out mainly occur. When the equivalent cutting area is the smallest, although the fiber will also develop compression-induced fracture and fiber pull-out, more resin matrix is smeared on the machined surface, so a better surface roughness value is obtained.

The functional relationship between surface roughness and equivalent cutting area is established by using a nonlinear fitting method, as follows:

(7) $$0{}^{\circ}\;\mathrm{PCD}\;\mathrm{tool}:\;Ra=2.679\times {\left(a_{p}\;a_{e}\;\frac{V_{f}}{V_{c}}\right)}^{0.318}\;\;\;\;\;\;\;R^{2}=0.956$$

(8) $$4{}^{\circ}\;\mathrm{PCD}\;\mathrm{tool}:\;Ra=5.047\times {\left(a_{p}\;a_{e}\;\frac{V_{f}}{V_{c}}\right)}^{0.517}\;\;\;\;\;\;\;R^{2}=0.925$$

### 3.3. Analysis of Variance (ANOVA) of Milling Force and Surface Roughness

The statistical significance factors of cutting force and surface roughness were analyzed by ANOVA. Table 4 and Table 5 show the contribution rate of cutting parameters to the cutting force of the 0° PCD tool and the 4° PCD tool, respectively. It can be seen from the table that the contribution rates of spindle speed to cutting force are the most significant, 41.12% and 53.16%, respectively, followed by feed speed, and the contribution rate of cutting depth is the smallest. Table 6 and Table 7 show the contributions of cutting parameters to the surface roughness of the 0° PCD tool and the 4° PCD tool, respectively. As can be seen from the table, the contribution rates of spindle speed to surface roughness are the most significant, 48.48% and 51.85%, respectively, followed by feed speed, and the contribution rate of cutting depth is the smallest. The conclusion is consistent with the experimental results obtained by Haddad M [38].

### 3.4. Delamination and Tearing

**Figure 10:**
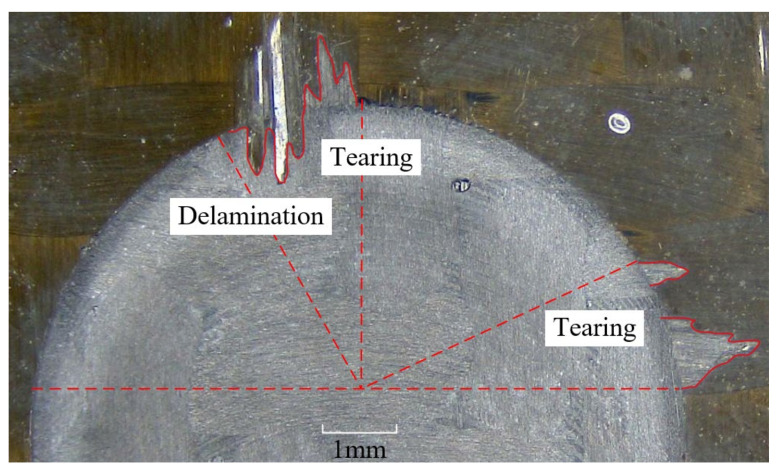
Delamination and tearing on top layer.

Delamination and tearing on the top layer, shown in Figure 10, were observed after milling. The warp fiber at the top of the groove develops delamination and tearing after milling. This is because the binding force between the carbon fiber on the upper surface layer and the matrix is weak, so some warp fibers will yield, only bending deformation will occur, and they cannot be cut by the milling cutter. If the strain force generated by bending deformation is less than the breaking strength of carbon fiber, the fiber will undergo elastic deformation after the tool leaves, resulting in delamination defects. The tearing defect is due to the rotation of the cutter, which stirs the uncut fiber into the cutting edge and produces tensile stress. When the tensile stress is greater than the tensile strength of the fiber, the fiber will break from the root, resulting in the tearing defect. Tearing of the weft yarn was also observed at the edge of the groove where the cutting tool was about to leave the cutting area. This is because the thickness of the uncut layer becomes smaller and smaller as the cutting edge goes from cutting into the workpiece to cutting out of the workpiece, and the support obtained by the surface fiber becomes smaller and smaller, so the fiber cannot be cut completely. Finally, the uncut fibers are torn off with the rotational movement of the tool, forming the tearing defects.

## 4. Conclusions

This paper studies the variation law of cutting force and surface roughness by milling a woven CFRP. The conclusions are as follows:

1. The increase in tool rake angle can improve the sharpness of the edge and play a positive role in reducing the cutting force. Additionally, the cutting force is positively correlated with the equivalent cutting area. When the equivalent cutting area increases, the cutting force increases. When the equivalent cutting area decreases, the cutting force also decreases.
2. The increase in tool rake angle has no obvious effect on surface roughness. However, the surface roughness will be affected by the equivalent cutting area, because the material-removal mechanism and failure form are different under different equivalent cutting areas.
3. The influence of spindle speed on cutting force and surface roughness is the most significant, followed by feed speed, and the influence of cutting depth is the least significant.
4. Delamination will appear on the top and both sides of the groove, which is due to the low restraint of the top fiber.

The results demonstrated that the equivalent cutting area has a significant effect on the cutting force and surface roughness when milling a CFRP. According to the research results, higher spindle speed, lower feed speed and cutting depth can be selected in actual production and processing. However, the total travel of the tool in this experiment was only 135 mm, and the wear of the PCD tool was not significant. It is recommended that tool life be further studied in future works.

## Figures and Tables

**Table 1:** Mechanical properties of the CFRP.

Tensile Strength/Mpa	Shear Strength/Mpa	Tensile Modulus/Gpa	Density/g·cm^−3^
4410	140	250	1.78

**Table 2:** Specific parameters of the tools.

	0° PCD Tool	4° PCD Tool
Diameter/mm	10	10
Number of cutting edge	2	2
Rake Angle/°	0	4
Clearance angle/°	13	13
Helix angle/°	0	0

**Table 3:** Taguchi’s experimental design, L9 orthogonal array with levels of selected parameters.

Experimental No.	Cutting Parameters			$\mathrm{Equivalent}\;\mathrm{Cutting}\;\mathrm{Area}\;\bar{A}$
	Spindle Speed n (rpm)	$\mathrm{Feed}\;\mathrm{Rate}\;V_{f}\;(m/\min)$	$\mathrm{Depth}\;\mathrm{of}\;\mathrm{Cut}\;a_{p}\;(\mathrm{mm})$	
1	6000	0.6	0.8	0.025465
2	6000	0.8	1	0.042441
3	6000	1	1.2	0.063662
4	8000	0.6	1	0.023873
5	8000	0.8	1.2	0.038197
6	8000	1	0.8	0.031831
7	10,000	0.6	1.2	0.022918
8	10,000	0.8	0.8	0.020372
9	10,000	1	1	0.031831

**Table 4:** ANOVA analysis for cutting force of 0° PCD tool.

Source	DOF	Seq.SS	Adj.MS	F	Contribution
Spindle speed	2	231.258	115.629	26.43	41.12%
Feed Rate	2	226.145	113.072	25.84	40.21%
Depth of cut	2	105.022	52.511	12.00	18.67%
Error	2	8.751	4.375		
Total	8	571.175			

**Table 5:** ANOVA analysis for cutting force of 4° PCD tool.

Source	DOF	Seq.SS	Adj.MS	F	Contribution
Spindle speed	2	183.475	91.7373	478.32	53.16%
Feed Rate	2	111.173	55.5864	289.83	32.21%
Depth of cut	2	50.498	25.2491	131.65	14.63%
Error	2	0.384	0.1918		
Total	8	345.529			

**Table 6:** ANOVA analysis for surface roughness of 0° PCD tool.

Source	DOF	Seq.SS	Adj.MS	F	Contribution
Spindle speed	2	0.04455	0.02228	67.35	48.48%
Feed Rate	2	0.03277	0.01638	49.54	35.66%
Depth of cut	2	0.01457	0.00728	22.03	15.86%
Error	2	0.00066	0.00033		
Total	8	0.09256			

**Table 7:** ANOVA analysis for surface roughness of 4° PCD tool.

Source	DOF	Seq.SS	Adj.MS	F	Contribution
Spindle speed	2	0.1176	0.0588	157.18	51.85%
Feed Rate	2	0.0577	0.0288	77.13	25.44%
Depth of cut	2	0.0515	0.0257	68.84	22.71%
Error	2	0.0007	0.0003		
Total	8	0.2275			

## Data Availability

Not applicable.
